# Engagement of chymase-positive mast cells in myocardial damage associated with COVID-19

**DOI:** 10.3389/fcvm.2026.1816510

**Published:** 2026-05-07

**Authors:** Andrey V. Budnevsky, Sergey N. Avdeev, Ekaterina D. Arkhipova, Djuro Kosanovic, Viktoria V. Shishkina, Tatiana A. Chernik, Evgeniy S. Ovsyannikov, Inna M. Perveeva, Andrey A. Filin, Roman E. Tokmachev, Alexander V. Pertsev, Elena E. Ivanova, Tatiana V. Samoylenko, Lyubov N. Antakova

**Affiliations:** 1 Department of Faculty Therapy, Burdenko Voronezh State Medical University, Voronezh, Russia; 2 Department of Pulmonology, I.M. Sechenov First Moscow State Medical University (Sechenov University), Moscow, Russia; 3 Department of Histology, Burdenko Voronezh State Medical University, Voronezh, Russia; 4 Scientific Research Institute of Experimental Biology and Medicine, Burdenko Voronezh State Medical University, Voronezh, Russia; 5 Department of Pathological Anatomy, Burdenko Voronezh State Medical University, Voronezh, Russia; 6 Voronezh Regional Pathoanatomical Bureau, Voronezh, Russia

**Keywords:** acute myocardial injury, cardiac fibrosis, chymase, COVID-19, mast cells

## Abstract

**Background:**

Acute myocardial injury is a frequent and severe complication of COVID-19, contributing to increased mortality. This study investigates the role of chymase-positive mast cells (MCs) in myocardial damage among patients who died from severe or critical COVID-19.

**Methods:**

Cardiac tissue samples from 60 deceased patients were analyzed histologically and immunohistochemically to assess MC density, degranulation status, and associated fibrotic remodeling. Clinical and laboratory parameters, including troponin I levels and inflammatory markers were obtained during hospitalization period.

**Results:**

The findings revealed a low overall density of cardiac MCs, with a predominance of degranulated chymase-positive cells. These MCs were significantly correlated with indicators of severe COVID-19 progression, such as neutrophilic leukocytosis, elevated band neutrophils, monocytosis, and increased erythrocyte sedimentation rate. A strong association was also observed between chymase-positive MCs and elevated troponin I levels, the association between troponin I and degranulating chymase-positive MC density remained statistically significant after multivariable adjustment, supporting an independent link to myocardial injury. Additionally, increased MC presence coincided with extensive interstitial and perivascular fibrosis, marked by a predominance of type III collagen.

**Conclusions:**

These results support the hypothesis that chymase-positive MCs contribute to myocardial inflammation and fibrosis in the context of COVID-19. Understanding their role may open avenues for targeted therapies aimed at mitigating cardiac complications in future coronavirus-related diseases.

## 1 Introduction

Acute myocardial injury is one of the possible coronavirus disease 2019 (COVID-19) complications. Its incidence varies widely and can range from 10.2% up to 69.2% in severe cases. The onset of acute myocardial injury in COVID-19 is associated with a poorer prognosis and an increased mortality risk (1).

The mechanisms of pathogenesis underlying heart damage in COVID-19 are diverse. Significant attention has been given to systemic inflammation, activation of the complement system and associated hypercoagulation. The ability of the SARS-CoV-2 virus to infect endothelial cells and induce endothelial dysfunction has also been demonstrated (2). Furthermore, evidence indicates the involvement of interleukin-6, which is highly expressed in COVID-19, in the development of myocardial dysfunction, ischemic complications and arrhythmias (3). Previously published data demonstrated the role of mast cell (MC) infiltration and degranulation in lung damage during COVID-19, as well as their contribution to hyperinflammatory responses (4, 5). Specifically, the relationships between MCs proteases (chymase, tryptase, and carboxypeptidase A3) in lungs and clinical markers of COVID-19 severity have been investigated (6, 7). However, the contribution of MCs to the inflammatory process in the heart and myocardial injury in COVID-19 remains poorly understood.

The aim of this study was to analyze the quantitative and functional features of chymase-positive mast cells, including their role in establishing a profibrotic microenvironment within the myocardium of patients who died due to COVID-19 and to assess their associations with clinical and laboratory parameters.

## 2 Materials and methods

Patients were recruited between September 2021 and June 2022. In the initial phase, data from 1,100 patients hospitalized with COVID-19 were analyzed. After applying the exclusion criteria, a preliminary sample was formed, which consisted of 754 patients.

The exclusion criteria included: chronic lung diseases (asthma, COPD, occupational lung diseases), pulmonary embolism, chronic heart failure with signs of systemic congestion, diabetes mellitus, hepatitis, liver cirrhosis, chronic kidney disease (CKD stage ≥ C3), active or history of malignant neoplasms.

The final sample was selected based on the following inclusion criteria: written informed consent to participate in the study, confirmed diagnosis of severe or critical COVID-19 (via polymerase chain reaction), in-hospital death due to acute respiratory distress syndrome (ARDS) verified by the Berlin definition criteria (8). Non-fulfillment of the inclusion criteria resulted in patient exclusion.

Objective patient data and laboratory parameters (complete blood count, biochemical blood analysis, C-reactive protein and procalcitonin) were collected. These parameters were registered on the first day of hospitalization and prior to the lethal outcome (the last pre-mortem measurements). Additionally, the concentration of troponin I was recorded once during the inpatient treatment period. The selection of treatment algorithms for patients was based on federal clinical guidelines, where the main pharmacological groups were glucocorticosteroids and anticoagulants. Interleukin-6 inhibitors were administered to 24 patients (40.0%), janus kinase inhibitors to 2 patients (3.33%) and anti-interleukin-6 monoclonal antibody therapy to 2 patients (3.33%). Convalescent plasma was also administered to 2 patients (3.33%).

During autopsy of deceased patients included in the final cohort, sections of the left ventricular myocardium cardiac tissues were collected for histological and immunohistochemical analysis. Tissue areas were selected without prior morphological preselection. Following fixation in 10% neutral buffered formalin and standard processing, serial sections of 4 μm thickness were prepared for Picro Mallory staining, while 2 μm sections were used for immunohistochemical staining of MC chymase, caspase 3 activity, and Picrosirius Red Staining to evaluate profibrotic components and assess the maturity of myocardial fibrosis. Separate serial myocardial sections were prepared for each histological and immunohistochemical staining procedure. Chymase-positive MCs were quantified morphometrically across 30 fields of view, accounting for their degranulation activity within the analyzed area, with results standardized per 1 mm^2^ (6). MC counting within the field of view was performed using QuPath software under visual supervision. Polarization microscopy was employed to quantitatively and qualitatively assess fibrillogenesis, distinguishing collagen type I (mature, appearing in red-orange birefringence) and type III (reticular, exhibiting green birefringence) fibers. Sections stained with Sirius Red were analyzed at 400× magnification using ImageJ software.

### 2.1 Statistical analysis

Statistical analysis was performed using Statgraphics Centurion 18. Qualitative variables are expressed as percentages and absolute numbers. Quantitative data are presented as mean ± standard deviation for normally distributed variables or median and interquartile range for non-normally distributed variables. Missing data were handled by complete-case analysis (listwise deletion) for each statistical test due to their low proportion (<5% for any variable). Primary data on MC density and troponin I concentration had no missing values. To compare clinical and laboratory parameters between the first day of hospitalization and the day before death, the paired Student's t-test (for normal distributed data) and the Wilcoxon signed rank test (for non-normal distribution) were applied. To account for multiple comparisons and multiple testing in the correlation analysis, *p*-values were additionally evaluated using the Benjamini–Hochberg false discovery rate procedure. Correlation analysis was performed using Spearman's correlation coefficient. Multivariable linear regression analysis was performed to assess the independent association between degranulating chymase-positive MC density and troponin I levels, adjusting for age, sex, hypertension, prior myocardial infarction, obesity, and key treatments (IL-6 inhibitors, unfractionated heparin, low-molecular weight heparin, and dexamethasone). Standardized *β* coefficients with 95% confidence intervals (CI) and *p*-values were reported. Statistical significance for group differences and correlations was at *p* < 0.05. The study was approved by the local ethics committee of the Burdenko Voronezh State Medical University (protocol №8, September 17, 2021).

## 3 Results

The final sample comprised 60 patients with confirmed severe or critical COVID-19 who died due to ARDS. Of these, 46.7% (*n* = 28) were female and 53.3% (*n* = 32) were male. The median age of the cohort was 70.0 [62.0; 72.0] years.

Common comorbidities included arterial hypertension (76.7%), stable angina (1.7%), postinfarction cardiosclerosis (1.7%), prior acute cerebrovascular accident (8.33%), obesity (15.0%), chronic heart failure without signs of systemic congestion (20.0%), chronic pyelonephritis (5.0%), and chronic kidney disease (CKD stage C1-C2) (6.67%). Clinical and laboratory parameters of the cohort are summarized in the Table 1. A trend toward progressive clinical deterioration was observed, including neutrophilic leukocytosis with elevated band neutrophils, lymphopenia, reduced hemoglobin and erythrocyte level, impaired renal function and coagulopathy. However, after Benjamini–Hochberg false discovery rate correction for multiple comparisons, statistical significance was retained only for comparisons with raw *p*-values ≤ 0.008.

**Table 1:** Clinical and laboratory parameters of patients with severe and critical COVID-19 (*n* = 60).

Parameters	On the 1st day of inpatient treatment	The last pre-mortem measurements	*p*
Objective clinical findings			
Heart rate, bpm	87.17 ± 12.23	88.4 ± 18.66	0.665
Respiratory rate, in min	24.0 [21.5; 24.0]	19,5 [18,0; 21,0]	**<0**.**001**
SBP, mmHg	130.27 ± 23.68	122.48 ± 23.44	**0**.**043**
DBP, mmHg	79.93 ± 13.15	75.92 ± 12.08	0.059
Complete blood count			
Hemoglobin, g/L	127.22 ± 26.11	122.29 ± 27.42	**0**.**008**
Erythrocytes, *10^12^/L	4.32 ± 0.84	4.04 ± 0.89	**<0**.**001**
Thrombocytes, *10^9^/L	176.5 [131.0; 195.0]	174.0 [121.0; 238.0]	0.234
Leukocytes, *10^9^/L	8.84 ± 5.01	14.34 ± 9.03	**<0**.**001**
Band neutrophils, %	5.07 ± 2.53	8.11 ± 4.48	**<0**.**001**
Basophils, %	0.0 [0.0; 0.0]	0.0 [0.0; 0.0]	0.891
Eosinophils, %	1.0 [0.0; 1.0]	1.0 [0.0; 1.0]	0.669
Lymphocytes, %	9.75 [5.0; 21.0]	5.0 [3.0; 10.0]	**<0**.**001**
Monocytes, %	5.0 [4.0; 8.0]	4.0 [3.0; 6.0]	0.284
ESR, mm/hour	30.5 [20.0; 52.0]	22.0 [6.0; 37.5]	**0**.**005**
Biochemical blood analysis			
Total protein, g/L	63.0 [56.3; 68.8]	56.0 [52.3; 65.0]	**<0**.**001**
Glucose, mmol/L	6.1 [5.8; 7.9]	6.7 [5.8; 8.8]	**0**.**033**
Total bilirubin, mmol/L	9.0 [6.0; 14.0]	9.5 [8.0; 13.0]	0.578
AST, IU/L	42.0 [32.5; 53.0]	36.5 [21.5; 49.0]	**0**.**042**
ALT, IU/L	30.5 [20.5; 52.0]	29.0 [21.5; 53.0]	0.884
Creatinine, µmol/L	114.0 [94.0; 141.5]	127.0 [107.8; 186.0]	**0**.**003**
Urea, mmol/L	7.4 [5.7; 11.8]	15.0 [8.2; 22.0]	**<0**.**001**
LDH, IU/L	841.0 [603.0; 1,519.3]	918.0 [704.0; 1,367.0]	**0**.**008**
APTT, mmol/L	38.3 [30.7; 45.85]	49.5 [33.3; 79.0]	**0**.**002**
INR	1.1 [0.89; 1.16]	1.48 [1.0; 2.2]	**0**.**005**
Fibrinogen, g/L	4.7 [4.2; 5.57]	4.2 [3.0; 5.5]	**0**.**044**
Markers of inflammation and myocardial necrosis			
CRP, mg/L	82.0 [36.3; 147.8]	113.0 [8.0; 192.0]	0.214
Procalcitonin, ng/mL	0.5 [0.0; 5.0]	2.0 [0.5; 10.0]	0.074
Troponin I, ng/mL	0.1 [0.0; 0.2]		**-**

Data with a normal distribution are presented as mean and standard deviation; data with a non-normal distribution are presented as median and interquartile range. ALT, alanine aminotransferase; APPT, activated partial thromboplastin time; AST, aspartate aminotransferase; CRP, C-reactive protein; DBP, diastolic blood pressure; ESR, erythrocyte sedimentation rate; INR, international normalized ratio; LDH, lactate dehydrogenase; SBP, systolic blood pressure.

Values with *p* < 0.05 are shown in bold.

Upon hospital admission 16.7% (*n* = 10) of patients had C-reactive protein (CRP) levels within the reference range, while 35% (*n* = 21) exhibited CRP levels exceeding 100 mg/L. In the last pre-mortem measurement, CRP levels were below 20 mg/L in 26.7% (*n* = 16) of patients, and exceeded 100 mg/L in 43.3% (*n* = 26).

Troponin I was measured once during the study, with 11.7% (*n* = 7) of the cohort showing values above the reference range.

The results of the immunohistochemical analysis of cardiac tissues revealed a predominance of chymase-positive MCs exhibiting degranulation and are presented in the Table 2.

**Table 2:** Chymase-positive mast cells density in cardiac tissues.

Subgroups of mast cells	Density per 1 mm^2^
Degranulated mast cells	0.17 [0.00; 0.77]
Non-degranulated mast cells	0.00 [0.00; 0.00]
Total number of mast cells	0.17 [0.00; 0.85]

Data are presented as median and interquartile range.

Correlations between the number of cardiac chymase-positive MCs and other assessed parameters were calculated. The results are presented in the Table 3.

**Table 3:** Correlations between chymase-positive cardiac MCs, clinical and laboratory parameters.

Parameters	CPMCs D^+^		CPMCs D^−^		Total CPMCs	
	r	*p*	r	*p*	r	*p*
Objective clinical findings						
Final recorded heart rate	**0**.**27**	**0**.**041**	**0**.**49**	**<0**.**001**	**0**.**30**	**0**.**021**
Final recorded respiratory rate	**0**.**28**	**0**.**030**	0.23	0.076	**0**.**29**	**0**.**026**
Final recorded SBP	**−0**.**34**	**0**.**010**	−0.17	0.181	**−0**.**34**	**0**.**010**
Final recorded DBP	**−0**.**32**	**0**.**013**	−0.11	0.378	**−0**.**33**	**0**.**011**
Complete blood count						
Hemoglobin at admission	**−0**.**30**	**0**.**030**	−0.18	0.196	**−0**.**29**	**0**.**036**
Erythrocytes at admission	**−0**.**40**	**0**.**005**	**−0**.**35**	**0**.**013**	**−0**.**39**	**0**.**005**
Pre-mortem erythrocytes	**−0**.**32**	**0**.**021**	**−0**.**31**	**0**.**027**	**−0**.**33**	**0**.**020**
Leukocytes at admission	**0**.**53**	**0**.**017**	**0**.**69**	**0**.**002**	**0**.**56**	**0**.**012**
Band neutrophils at admission	**0**.**790**	**<0**.**001**	**0**.**58**	**0**.**009**	**0**.**80**	**<0**.**001**
Pre-mortem band neutrophils	**0**.**49**	**0**.**028**	**0**.**45**	**0**.**042**	**0**.**53**	**0**.**018**
Pre-mortem basophils	**−0**.**33**	**0**.**016**	−0.06	0.656	**−0**.**29**	**0**.**034**
Pre-mortem monocytes	**0**.**34**	**0**.**014**	0.27	0.054	**0**.**36**	**0**.**010**
ESR at admission	**0**.**32**	**0**.**017**	0.14	0.293	**0**.**32**	**0**.**017**
Biochemical analysis						
LDH at admission	**−0**.**39**	**0**.**034**	−0.16	0.369	**−0**.**36**	**0**.**050**
APTT at admission	**0**.**35**	**0**.**007**	**0**.**34**	**0**.**009**	**0**.**37**	**0**.**005**
INR at admission	**−0**.**65**	**0**.**002**	−0.36	0.096	**−0**.**65**	**0**.**002**
Pre-mortem INR	**−0**.**70**	**0**.**008**	−0.44	0.101	**−0**.**70**	**0**.**009**
Fibrinogen at admission	**−0**.**34**	**0**.**028**	**−0**.**36**	**0**.**017**	**−0**.**37**	**0**.**015**
Pre-mortem fibrinogen	**−0**.**38**	**0**.**021**	**−0**.**45**	**0**.**006**	**−0**.**40**	**0**.**015**

APPT, activated partial thromboplastin time; CPMCs, chymase-positive mast cells; D^+^, with degranulation; D^−^, without degranulation; DBP, diastolic blood pressure; ESR, erythrocyte sedimentation rate; INR, international normalized ratio; LDH, lactate dehydrogenase; SBP, systolic blood pressure.

Values with *p* < 0.05 are shown in bold.

**Figure 1:**
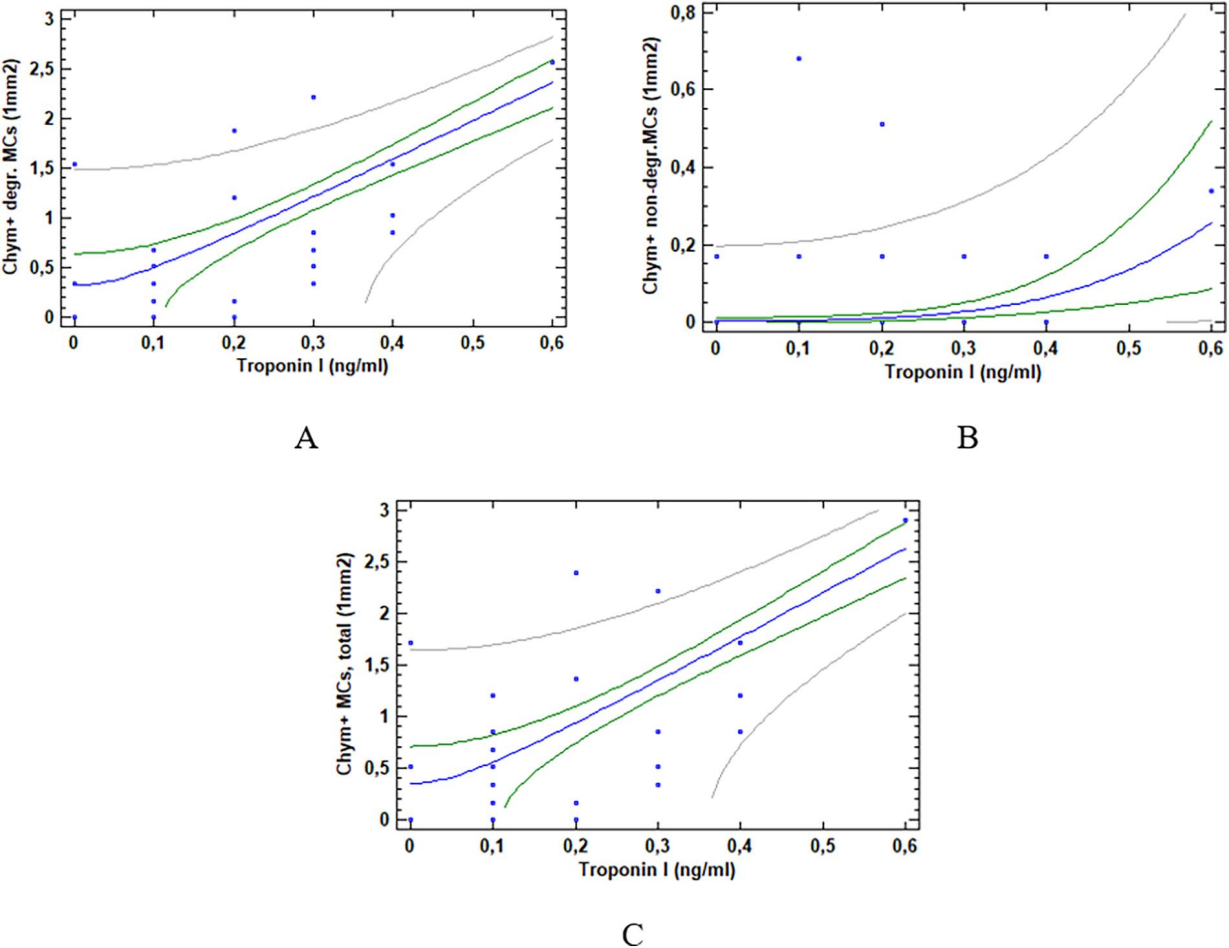
Correlations between chymase-positive cardiac MCs and troponin I. **(A)** Correlation between degranulated mast cells and troponin I. **(B)** Correlation between non-degranulated mast cells and troponin I. **(C)** Correlation between total number of mast cells and troponin I. The plots show least squares regression lines (blue) and two sets of limits. The inner limits (green) represent 95% confidence intervals for the mean value of Y at any selected X. The outer lines (grey) are 95% prediction limits for new observations.

Furthermore, significant correlations were identified between the investigated subgroups of chymase-positive mast cells, the blood procalcitonin concentration at admission and serum troponin I level. The correlation coefficients and significance levels are presented in Table 4. After Benjamini–Hochberg false discovery rate correction for multiple testing in Tables 3, 4, statistical significance was retained for correlations with raw *p*-values ≤ 0.0363.The relationships between chymase-positive mast cells and troponin I are graphically illustrated by regression lines in Figure 1.

**Table 4:** Correlations between chymase-positive cardiac MCs, markers of inflammation and myocardial necrosis.

Parameters	CPMCs D^+^		CPMCs D^−^		Total CPMCs	
	r	*p*	r	*p*	r	*p*
Procalcitonin at admission	**0**.**67**	**0**.**010**	**0**.**58**	**0**.**025**	**0**.**67**	**0**.**010**
Troponin I	**0**.**67**	**<0**.**001**	**0**.**31**	**0**.**017**	**0**.**66**	**<0**.**001**

CPMCs, chymase-positive mast cells; D^+^, with degranulation; D^−^, without degranulation.

Values with *p* < 0.05 are shown in bold.

Multivariable linear regression analysis demonstrated that degranulating chymase-positive MCs density was independently associated with troponin I levels after adjustment for age, sex, hypertension, prior myocardial infarction, obesity, and key treatments (IL-6 inhibitors, unfractionated heparin, low-molecular weight heparin, dexamethasone). The association remained statistically significant (*β* = 0.633, 95% CI 0.418–0.847, *p* < 0.001), indicating that the relationship between MCs and myocardial injury is not confounded by these clinical and therapeutic factors.

**Figure 2:**
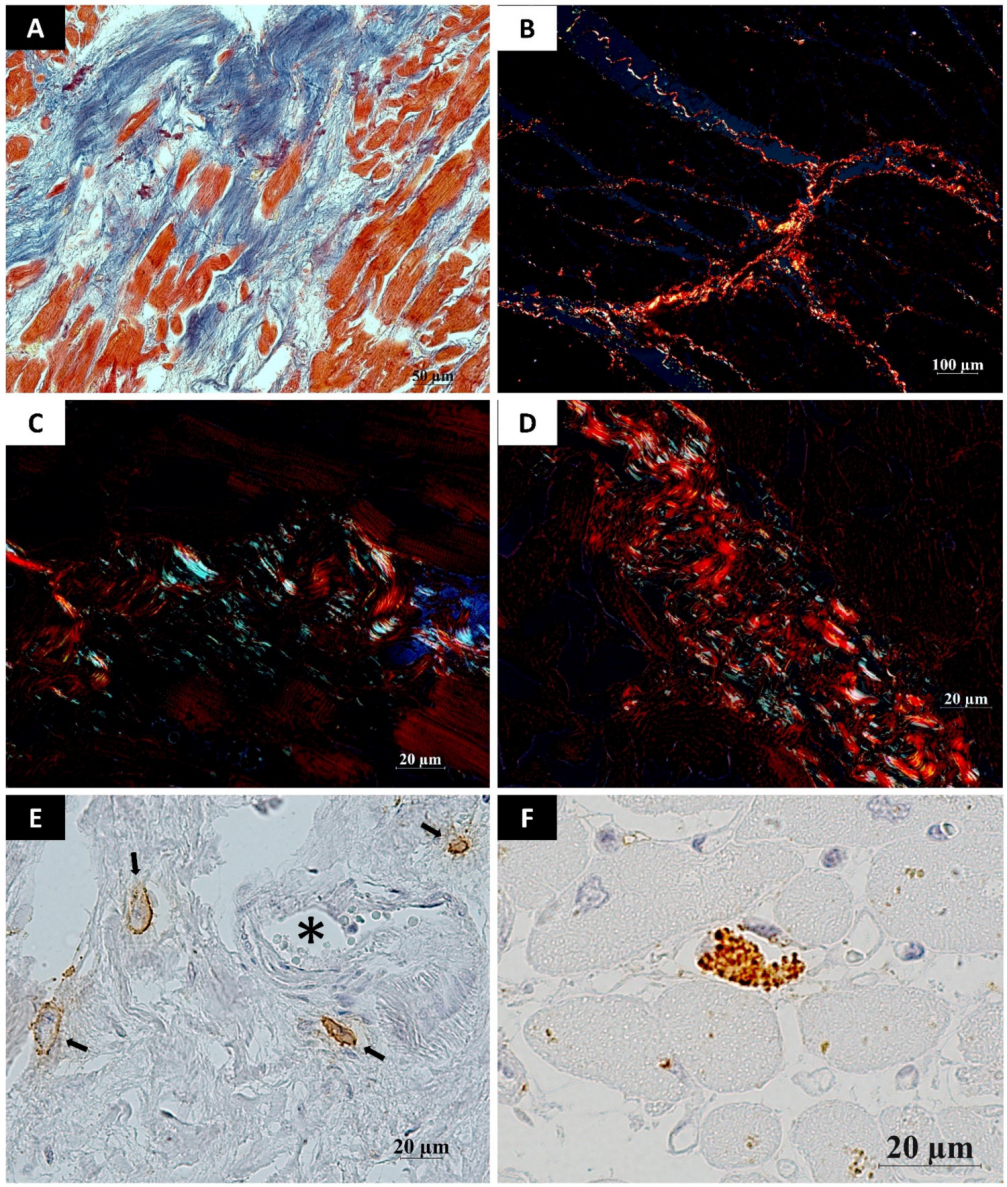
Features of myocardial stromal remodeling in COVID-19 patients. **(A)** Myocardial tissue exhibiting fibrotic foci (blue). Cardiomyocytes show atrophy and hypertrophy, separated by thin fibrous septa. Single atrophic cardiomyocytes are embedded within the fibrotic connective tissue; **(B)** Connective tissue fibers of varying maturity dissecting cardiomyocytes bundles. Mature type I collagen fibers predominate (red, yellowish-orange birefringence under polarized light). Green birefringence corresponds to type III collagen, with its immature fibers being less abundant in the connective tissue; **(C,D)** Areas of myocardial fibrosis. The connective tissue is heterogeneous: some regions exhibit predominate bundles of type III collagen fibers (green birefringence), while others are dominates by type I collagen fibers (red birefringence); **(E)** Mast cells (arrows) near the blood vessel (*) in foci of fibrosis in the myocardium. Chymase–positive granules are located on the periphery of the cytoplasm, a sign of degranulation of MC; **(F)** A mast cell situated between cardiomyocytes. Cytoplasmic granules exhibiting signs of exocytosis are distinctly visible within the mast cell. **Techniques:** A – Mallory method, B-D – staining with Picrosirius red, E,F – IHC-reaction to detection of mast cell chymase (mouse monoclonal anti-mast cell chymase antibodies (AbCam, #ab2377, dilution 1:2,000). Scale bar: A – 50 µm, B – 100 µm, C-F – 20 µm.

**Figure 3:**
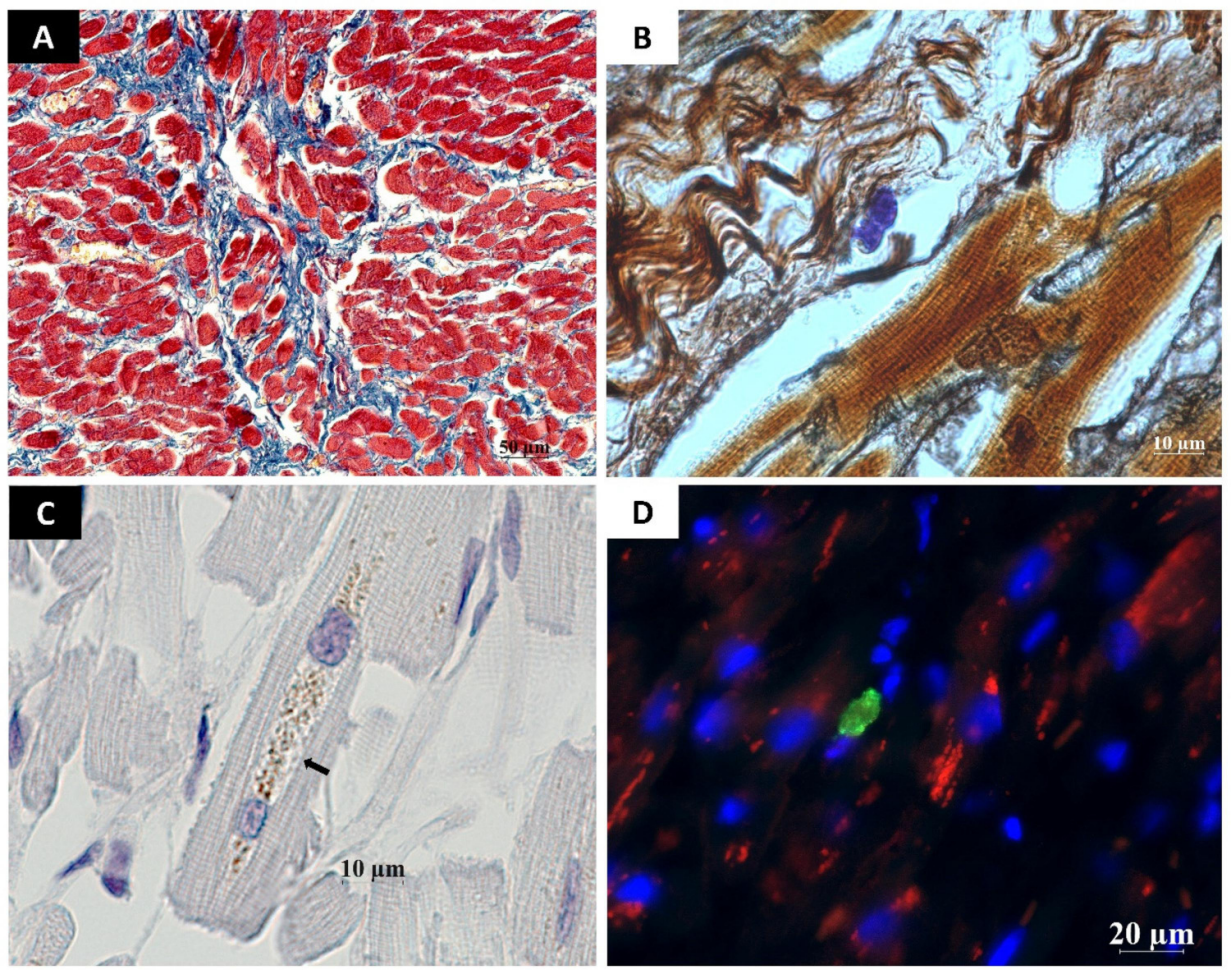
Some features of cellular reactivity of myocardial tissues in COVID-19 patients. **(A)** Area of myocardial fibrosis. Thin layers of connective tissue (blue) separate fascicles of cardiomyocytes with signs of hypertrophy and atrophy; **(B)** Reticular fibers in a fibrotic area of the myocardium (black). In the center of the area is a mast cell closely interacting with the fibers. Silver impregnation, toluidine blue; **(C)** Cardiomyocyte with intraplasmic accumulation of Caspase 3+ granules (arrow). The granules are characteristically located at the edges of the nuclei (periapically); (**D**) Colocalization of Chymase+ mast cells and Caspase+ granules in myocardial cells. **Techniques:** A – Picro-Mallory stain method, B – combined method of silver impregnation and toluidine blue, C – IHC with primary rabbit polyclonal antibodies to Caspase 3 (AbCam, #ab4051, dilution 1:500); D – immunofluorescence IHC method, double labeling with antibodies to Chymase and Caspase 3. Scale bar: A – 50 µm, B,C – 10 µm, D – 20 µm.

Patients with high MC chymase levels had increased reposition of collagen types I and III. Two forms of reactive fibrosis have been described. The first form is characterized by an abundance of collagen fibers and interstitial localization. The second form is distinguished by perivascular localization of fibrosis and a greater number of inflammatory cells (9). In our study, perivascular and interstitial fibrosis coexisted in heart samples where high chymase density was noted (Figure 2). The area of fiber fluorescence in the myocardial tissue during fibrosis development ranged from 8% to 32% of the analyzed area. In COVID-19, the level of active Caspase—3 in cardiomyocytes is significantly elevated, driving extrinsic apoptotic pathway and activates cardiomyocyte apoptosis (Figure 3). Mast cell chymase can degrade extracellular matrix survival factors and directly process cytokines to activate the caspase cascade in the cells.

## 4 Discussion

The heart is an organ with a relatively low concentration of MCs under physiological conditions. Most studies reporting MC density in cardiac tissues derive from animal models, while clinical data in humans remain scarce. According to the study by Janicki J.S. et al., the average cardiac MC density was 5.3 cells/mm^2^ across all MC phenotypes (10). Though chymase-positive MCs were not specifically quantified. In COVID-19 patients receiving anti-inflammatory therapy, the observed low MC density in cardiac samples may reflect therapy-induced MC suppression. Conversely, cardiac stress (due to ischemia, pressure/volume overload) is associated with increased MC density (10).

The biological effects of chymase on surrounding tissues are diverse and include angiotensin (AT) I hydrolysis to AT II, endothelial apoptosis, proinflammatory cytokines activation, atherosclerotic plaques destabilization, fibrosis and amplification of MC degranulation (11). Our findings revealed negative correlations between chymase-positive MC counts and pre-mortem systolic and diastolic blood pressure. This aligns with evidence that chymase-derived AT II minimally affects systemic blood pressure (12), instead promoting local inflammation, fibrosis and pathological myocardial hypertrophy (13). Thus, proinflammatory cytokines activation, increased production of reactive oxygen species and fibrosis may contribute to the development of systolic dysfunction and hypotension. In addition, both the MC activation and hypotension are manifestations of a systemic inflammatory process. Developing hypotension, systolic dysfunction, as well as reduced erythrocytes and hemoglobin shown in this study could collectively drive compensatory tachycardia. Furthermore, AT II enhances norepinephrine release and sympathetic tone, potentially explaining its proarrhythmogenic effects (10).

In our study, we identified associations between an increase in chymase-positive MCs and parameters indicative of the severity of COVID-19: leukocytosis with elevated band neutrophil counts, monocytosis, increased erythrocyte sedimentation rate, and reduced basophil counts. It is known that degranulation of chymase-positive MCs triggers the migration of neutrophils, lymphocytes, and monocytes, thereby perpetuating a pro-inflammatory microenvironment within specific tissue loci (14). Furthermore, this protease has been shown to promote leukocyte adhesion to vascular endothelium via cytokine production and AT II generation (15).

In severe COVID-19, coagulation disorders such as disseminated intravascular coagulation may occur, characterized by hypercoagulable and hypocoagulable phases. During hospitalization, the study cohort exhibited significant increases in activated partial thromboplastin time (APTT) and international normalized ratio (INR), along with decreased fibrinogen levels, though mean fibrinogen values remained above reference ranges. The most pronounced differences were observed in APTT, likely due to heparin use in COVID-19 treatment. The negative correlation between chymase-positive MC counts and fibrinogen may reflect chymase's ability to cleave fibrinogen and enhance its binding to platelets (16, 17). Fibrinogen cleavage might also contribute to prolonged APTT. The divergent correlations of chymase-positive MCs with APTT and INR may indicate predominant depletion of intrinsic coagulation pathway factors alongside activation of the extrinsic pathway via tissue factor synthesis stimulation (18).

Assessment of troponin I levels in patients with severe or critical COVID-19 revealed elevated levels in 11.7% of cases, indicating acute myocardial injury. Positive correlations were observed between all chymase-positive MC subgroups and troponin I concentrations; importantly, the association between troponin I levels and degranulating chymase-positive MC density remained significant after adjustment for relevant confounders in multivariable analysis, supporting the independent relationships of this MC phenotype with myocardial injury. Existing evidence shows increased chymase-positive MC density in areas of cardiomyocyte ischemic damage, while chymase inhibition reduces necrotic zones, arrhythmia incidence and mortality (19, 20). MCs are among the primary sources of fibroblast growth factors and transforming growth factor-β, the key fibrogenic factor in cardiac tissue. Cardiac fibrosis represents the terminal stage of a dynamic tissue remodeling process involving multicellular interactions and the activation of numerous immune cells, including MCs, macrophages, neutrophils, eosinophils, and T-cell subsets.

It is important to note that the role of MCs in cardiac injury is dual. The beneficial effects of MC-derived chymase are manifested during tissue healing and scar formation through the stimulation of collagen fibril production and the activation of vascular endothelial growth factors (VEGF-A and VEGF-C) in the vascular and cardiac lymphatic systems (10). This may enhance inflammation resolution and play a critical role in mitigating myocardial edema. Thus, MC-derived chymase may exert potential cardioprotective effects.

Myocardial injury in severe COVID-19 arises as part of a complex inflammatory cascade and cannot be explained by MC-related changes alone. In this setting, chymase-positive MCs should be considered one of the cellular components contributing to cardiac injury rather than an isolated mechanism. Importantly, even a relatively low myocardial MC density does not exclude biological relevance, since activated or degranulating chymase-positive MCs may exert substantial local proinflammatory and profibrotic effects. Their association with elevated troponin I levels, hypotension, and coagulation abnormalities supports the clinical relevance of these histological findings.

## 5 Limitations

The study did not analyze cardiac tissue samples from healthy individuals or patients with non-fatal COVID-19. This limits the interpretation of whether the observed alteration in MC density and degranulation activity are specific to fatal COVID-19. Comparative analysis of MC density and degranulation activity between healthy heart tissues and heart tissues samples from COVID-19-infected patients could provide deeper insights into the role of MCs in the development of cardiac injury in COVID-19 patients.

## 6 Conclusion

Acute myocardial injury was observed in 11.7% of patients with severe and critical COVID-19. During hospitalization, the study cohort exhibited a significant decrease in respiratory rate, systolic and diastolic blood pressure, hemoglobin levels, erythrocytes, lymphocytes, and ESR, alongside an increase in leukocytes and band neutrophils. Serum biochemical parameters indicated impaired renal function and hypocoagulation.

The density of chymase-positive MCs in cardiac tissues was 0.17 [0.00; 0.85] per mm^2^, with a predominance of cells exhibiting degranulation. Correlation analysis identified associations between chymase-positive MCs and several markers reflecting severe COVID-19 progression: neutrophilic leukocytosis with an elevated proportion of immature forms, monocytosis, and increased ESR. Direct correlations were also observed between chymase-positive cardiac MCs and troponin I levels, indicating an association between chymase-positive MCs and myocardial injury, although a causal relationship cannot be established from this study.

The increase in chymase expression was accompanied by remodeling processes of the fibrous components of the cardiac extracellular matrix, predominantly marked by elevated levels of type III collagen. Myocardial fibrosis is a multifactorial process involving inflammation, immune responses, tissue damage, pressure overload and other factors. Current treatments for myocardial fibrosis remain limited by numerous challenges. However, accumulated data on the association between MC chymase and myocardial remodeling indicate the need for further studies to evaluate the potential therapeutic significance of this mechanism.

## Data Availability

The raw data supporting the conclusions of this article will be made available by the authors, without undue reservation.
